# Development and Assessment of a Soft Wearable for sEMG-Based Hand Grip Detection and Control of a Virtual Environment

**DOI:** 10.3390/s25082431

**Published:** 2025-04-12

**Authors:** Lohith Chatragadda, Aiden Fletcher, Sam Zhong, Fabian A. Vargas, Nishtha Bhagat, Kunal Mankodiya, Matthew J. Delmonico, Dhaval Solanki

**Affiliations:** 1 Interdisciplinary Neuroscience Program, University of Rhode Island, Kingston, RI 02881, USA; lchatragadda@uri.edu; 2 Department of Kinesiology, University of Rhode Island, Kingston, RI 02881, USA; aiden_fletcher@uri.edu (A.F.); fabian_vargas@uri.edu (F.A.V.); delmonico@uri.edu (M.J.D.); 3 Department of Computer Science and Statistics, University of Rhode Island, Kingston, RI 02881, USA; shan_zhong@uri.edu; 4 Department of Electrical, Computer and Biomedical Engineering, University of Rhode Island, Kingston, RI 02881, USA; nishtha.bhagat@uri.edu (N.B.); kunalm@uri.edu (K.M.)

**Keywords:** electromyography, neurodegenerative disorder, rehabilitation, grip strength, grip force, virtual environment

## Abstract

Background: As the number of individuals diagnosed with neurodegenerative disorders (NDs) rises, there is a growing need to enhance both the quantity and quality of approaches used to treat these debilitating conditions. The progression of NDs can cause muscle weakness in the lower or upper limbs. We particularly focus on the area of the upper limb, specifically grip rehabilitation, by developing a system (VRGrip) that can reliably record electromyography (EMG) events of the hand flexor muscles to control an adaptive and engaging game using grip exertion. The purpose of this study was to determine the feasibility of using the VRGrip system. Methods: We prototyped a three-component wearable system consisting of an e-textile forearm band (E-band), data acquisition module (DAM), and a computer game. This allows participants to play a game by squeezing their dominant hand. A feasibility study was completed with 9 individuals who self-reported an ND (including Parkinson’s disease (PD), amyotrophic lateral sclerosis (ALS), multiple sclerosis (MS), Charcot–Marie–Tooth disease (CMT), spinal muscular atrophy (SMA), and essential tremor (ET)) and 12 individuals who self-reported to be relatively healthy (RH). Each participant completed 15 min of gameplay (three trials of five minutes), where they would squeeze a resistive ball to trigger in-game actions. The user experience was then evaluated via a User Satisfaction Evaluation Questionnaire (USEQ; scored 0–30, with 30 being best). Results: Analysis of the grip detection reliability during the feasibility study resulted in an F1 score of 0.8343 ± 0.1208 for the healthy participant group and 0.8401 ± 0.1034 for the ND participant group. The USEQ (Avg. score: 4.65 ± 0.51) indicated that participants found the system comfortable, engaging, and enjoyable. Additionally, we potentially identified age-related changes in muscle fatigue. Conclusion: The results of this study demonstrate that our VRGrip system could be used for hand grip detection in a virtual environment. In the future, we aim to conduct longitudinal studies to determine if repeated use of the system has merit for grip rehabilitation.

## 1. Introduction

Individuals with neurodegenerative disorders (NDs) such as Parkinson’s disease (PD), amyotrophic lateral sclerosis (ALS), or multiple sclerosis (MS) often experience a decline in muscular function and strength as the diseases progress [1]. One of the most functionally debilitating consequences of this muscle weakness is hand grip weakness; grip weakness is traditionally addressed through physical therapy, primarily focusing on strengthening the hand flexor and extensor muscles through repeatedly squeezing a resistive ball (flexion) and opening the fingers while resisting an elastic band (extension) [1,2]. These physical therapy regimens often combine occasional in-clinic sessions with a defined schedule of exercises to be performed at home. The main limitations of the at-home rehabilitation portion of these regimens are the (1) lack of accessible equipment, (2) limited methods to quantify and track grip rehabilitation progress over time, and (3) lack of adherence to exercise schedules [3]. Only around 21% of individuals who partake in at-home rehabilitation programs fully adhere to the prescribed rehabilitation plan; this lack of adherence will likely impede optimal strength improvements provided by the rehabilitation program [4]. Additionally, clinicians are often not aware of the extent of program adherence/non-adherence and often have to rely on patient reports about their progress on program [5].

Currently, assessment of grip rehabilitation progress is performed by periodically measuring grip force using a dynamometer [6]. While accurate in quantifying grip strength, these measurements are performed in the clinic, which inherently limits the frequency of such assessments. A holistic look at grip force, as provided by the grip dynamometer, also does not provide specific information about muscle function. The dynamometer cannot be useful in patients with limited grip ability who cannot grip the handle of the dynamometer [7]. The repetitive movement of the dynamometer can be monotonous as a rehabilitation exercise and can lead to non-adherence due to boredom.

Other researchers have explored building devices and engaging virtual environments for rehabilitation to increase user engagement and adherence [8,9,10]. For example, Zhang et al. designed and built a glove that allows users to perform grip rehabilitation in an engaging augmented reality (AR) environment [11]. Flex sensor technology has also been used to detect the angle of a user’s fingers to detect hand grip and interface with various digital rehabilitation tools [12]. Other researchers have also developed non-wearable devices that can detect grip force and allow for the rehabilitation of other functional hand movements like pinching, rolling, and squeezing [13]. 

In contrast to existing systems, we aimed to create a system that is both engaging for the user (patient side) and collect relevant data for longitudinal analysis (clinician side). The VRGrip system is a three-component system for hand grip detection, including (i) a soft e-textile forearm band (E-band), (ii) a data acquisition module (DAM), and (iii) an interactive game environment. The VRGrip system utilizes surface electromyography (sEMG), which measures the electrical signal generated by the contraction of a superficial muscle(s). In our system, activity of the flexor digitorum superficialis and flexor digitorum profundus muscles is recorded. Since these muscles are responsible for finger flexion, monitoring their sEMG activity allows users to control an engaging game environment by closing their fingers. Through this approach, we offer a direct look at muscle activity related to grip exertion, along with a method of interaction using this activity, which may offer a more useful look at the progression of muscle dysfunction.

The major contributions of our work include the following:

- Development of the VRGrip System: We designed an innovative e-textile forearm band (E-band) integrated with a data acquisition module (DAM) and an adaptive game engine. The system captures surface electromyography (sEMG) signals from hand flexor muscles, translates them into game controls, and provides a compact, wireless, and user-friendly interface suitable for rehabilitation purposes.
- Innovative Features for Engagement and Data Analysis: We created a virtual reality game environment with adaptive difficulty based on user performance to enhance engagement. We also developed a custom calibration algorithm for accurate sEMG-based grip detection and analyzed muscle metrics such as median frequency (MDF) and mean peak amplitude (MPA) to assess fatigue and muscle performance.
- Feasibility Study and User Evaluation: We conducted a feasibility study with participants from diverse health backgrounds (relatively healthy and individuals with neurodegenerative disorders). We demonstrated the system’s reliability in detecting grip exertion, analyzed sEMG data to explore muscle fatigue, and evaluated user satisfaction, identifying key insights for future system improvements.

## 2. Materials and Methods

### 2.1. Electronic Forearm Band (E-Band) Fabrication

**Figure 1:**
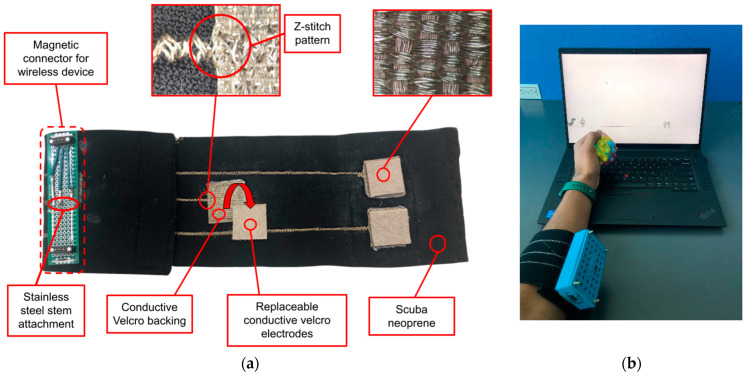
E-band prototype. (**a**) The conductive fabric electrodes are composed of two connecting pieces of conductive hook-and-loop tape. As the microscope image shows, the skin-contacting part of the electrode is a relatively flat surface of silver-coated polyester. (**b**) The right side of the figure shows the study setup with E-band being worn on the forearm.

The E-band is a textile-based forearm band [14,15] that uses conductive fabric patches as electrodes to detect sEMG signals (Figure 1). A 35.6 cm × 8.2 cm piece of scuba neoprene was used as the base material for the E-band. Then, 2.5 cm × 2.5 cm pieces of conductive hook tape were sewn onto the scuba neoprene using silver-coated polyester conductive thread as the top thread, and stainless steel thread as the bottom thread, in a “Y” orientation [16]. These fabric pieces formed the three electrodes/leads for sEMG acquisition of the hand flexor muscles. As seen in Figure 1, the two electrodes near the end of the band are designed to be positioned over the central and distal portions of flexor muscle bundles belly. The singular electrode near the middle was intended to contact the olecranon process (bony part of the elbow) and act as the reference electrode.

Following the attachment of the three electrodes, conductive traces were made, extending across the band using the conductive thread. The stitches were placed from the center of each electrode to a point near the opposite end of the band using a Z-stitch pattern (see Figure 1) to provide a robust electrical connection even when the band was stretched to accommodate varying sizes of users’ forearms. A small “stem” of stainless-steel thread was left hanging off the back of the band. This stem was used to connect the electrodes to a small piece of a circuit board for interfacing with the data acquisition module (DAM; explained in the following section). Each stem was saturated with stainless steel flux for five minutes and then covered with lead-free solder to create a wire-like connection to the circuit board (see Figure 1). Each of the stems was inserted into a separate hole of the circuit board, and the stems were connected using a lead-free solder. A three-pin magnetic connector was then soldered to each end of the circuit board, and 20 AWG wire was used to connect the three soldered stems to the pins of the magnetic connector. Through this process, an electrical connection was created between the conductive Velcro electrodes and the magnetic connector. 

We created a 2.5 cm × 2.5 cm square of conductive Velcro (loop side) and it was placed beneath the conductive electrode. The skin-contacting part of the electrodes have a relatively flat surface, which maximizes contact with the skin. These skin-contacting parts are also replaceable in the case of corrosion, which can extend the lifespan of the E-band. Specifically, when used for extended periods of time, sweat may cause corrosion of the silver-coated polyester that makes up the electrodes. In this case, users can remove the layer of Velcro that contacts the skin and easily replace it with a new patch of Velcro, which will restore good signal quality.

### 2.2. Data Acquisition Module (DAM)

**Figure 2:**
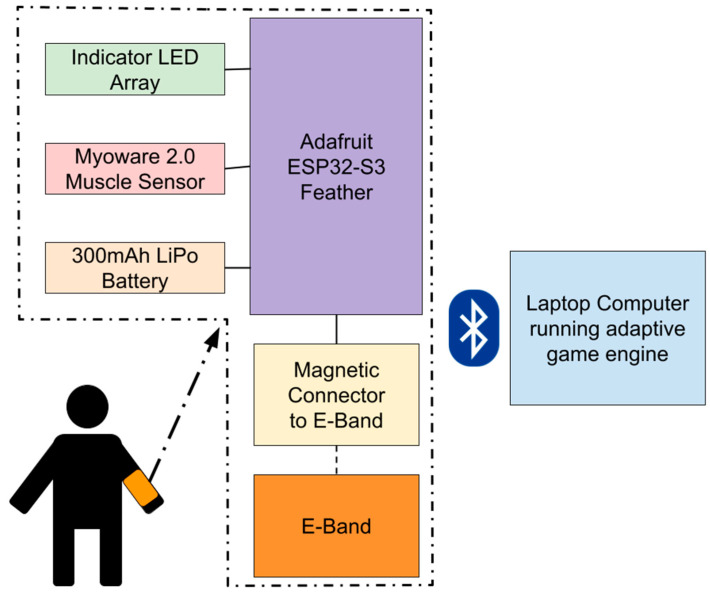
VRGrip system block diagram. The E-band on the participants forearm connects to the DAM, which is capable of wireless Bluetooth communication. It sends EMG data to a laptop computer at 500 samples per second, allowing users to control a computer game.

The data acquisition module (DAM) is the central hardware component of the VRGrip system, translating muscle activity into digital signals for the game engine (Figure 2). It acts as a bridge between the E-band and the adaptive game engine by (1) recording sEMG signal on the hand flexor muscles in the forearm (flexor digitorum superficialis and flexor digitorum profundus), using the E-band as electrodes, and (2) transmitting these data to a computer via Bluetooth to enable the end user to interact with the game.

**Figure 3:**
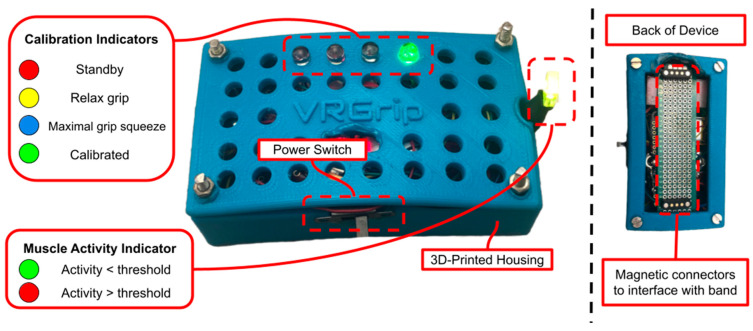
DAM diagram. The DAM features two magnetic connectors that allow the user to easily connect it to the E-band and begin calibration and gameplay.

As seen in Figure 2, the DAM uses a Bluetooth-enabled ESP32-S3 microcontroller (Adafruit Industries, NY, USA) interfaced with a Myoware 2.0 Muscle Sensor (Sparkfun Industries, Niwot, CO, USA) to perform sEMG; the solder pads associated with mid, end, and reference electrodes on the Myoware are connected to a magnetic connector that snaps onto the E-band [17,18]. Myoware was selected because it is relatively inexpensive, and has previously been shown to collect sEMG signals comparable to commercially available systems [19]. The device is also connected to a 3.7 V LiPo battery (PKCell Battery Co., Ltd., Shenzhen, China) with a capacity of 300 mAh to give it wireless capabilities. The DAM was then enclosed in a custom 3D-printed housing to conceal electronic components from users (Figure 3). 

Several indicator LEDs are interfaced with the ESP32-S3 to guide users through the automated calibration process. The calibration sequence includes four device states: standby state (S1), relax grip state (S2), maximal grip squeeze state (S3), and calibrated state (S4). The device states are indicated by the four colored LEDs visible on top of the DAM (refer to Figure 3 and Table 1). During S1, the red LED is illuminated to indicate that the user has five seconds to position the DAM onto the E-band using the magnetic connectors. Once five seconds have elapsed, the DAM will transition to S2, during which the yellow LED is illuminated, indicating that the user should relax their hand grip so that the device can calibrate to the amplitude of the sEMG signal at rest. Following five seconds of resting hand grip in S2, the DAM transitions to S3; the blue LED is illuminated, which indicates the user to squeeze their hand grip as hard as possible for three seconds. This allows the DAM to calibrate to the sEMG amplitude corresponding to maximal exertion by the user. Once this calibration occurs, the DAM transitions to S4. The illuminated green LED in this state indicates that the DAM has been calibrated to the user’s muscle activity and is ready for the user to control the computer game using their hand grip.

Once the calibration procedure is completed, the DAM begins to transmit the raw sEMG values and trigger values to a Bluetooth-connected laptop at a rate of 500 samples per second using Bluetooth communication. Additionally, the muscle activity indicator LED turns on after the calibration procedure is completed. A steady green color indicates that no significant muscle activity is being detected, and a red color indicates that muscle activity exceeding the calibrated threshold is being detected.

### 2.3. Software and Algorithms

The calibration procedure performed by the DAM relies on a custom algorithm that detects hand grip exertion from the raw sEMG data. During S2 of the calibration procedure, the DAM acquires an average amplitude of the collected raw sEMG data. In S3, the same metric is collected during maximal grip exertion. The percentage difference between these two averages is calculated and the threshold to trigger a click is then set to 60% difference between full relaxation and maximal contraction; this represents a moderate amount of grip effort that the users could repeatedly exert during gameplay. 

The amplitude of the signal is calculated on-board the ESP32-S3 by taking the absolute value of the difference between the latest EMG value and the previous value. A rolling average of the last 300 amplitude measurements is used during the calibration procedure to set the threshold for detected muscle activity. If the calibration procedure fails to reliably detect muscle activity, the gain of the Myoware 2.0 EMG sensor is adjusted using the built-in potentiometer.

**Figure 4:**
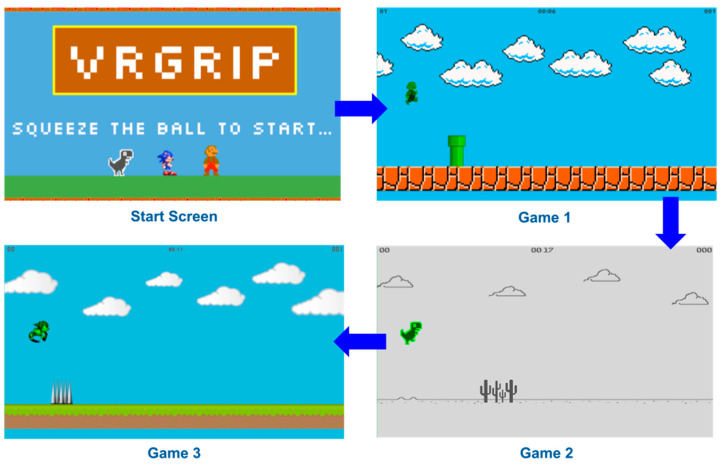
VRGrip custom game environment. Our game environment features three distinct games that are designed to be familiar to users of all populations. The custom game engine traverses difficulty levels based on user performance, and game switching is triggered by time elapsed.

The VRGrip software suite has two distinct software programs that work hand-in-hand. The first program is a handshaking script between the DAM and the laptop computer. This program accesses the Bluetooth serial port of the laptop that the DAM is connected to and collects the transmitted raw sEMG and trigger data. These data are stored in a spreadsheet for subsequent analysis. The program also interprets trigger data and generates a simulated keypress to the laptop. The second program is an adaptive game engine (see Figure 4; discussed in the next section) that responds to a single keypress (output by the first program). 

### 2.4. Game Development

**Figure 5:**
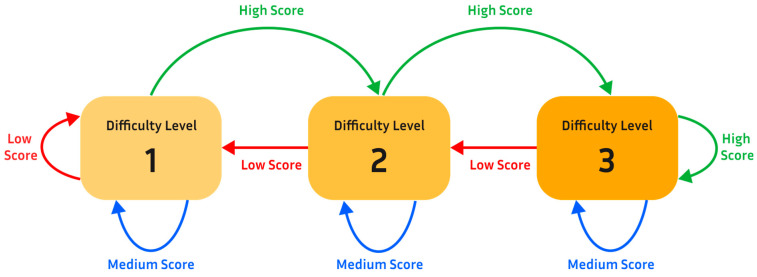
Game engine schematic. The game engine changes the difficulty level (DL) of the game based on a rolling record of the score (success rate of user jumps over past ~1 min). If the user performs very well in the measured segment, the game difficulty increases. If the user performs poorly, the difficulty level decreases. If the user performs moderately well, the difficulty level remains the same.

The game engine was developed in Unity 3.12f1 using C# [20]. The VRGrip game engine features three games that are simple yet engaging for users (see Figure 4). The objective of each game was similar: the user causes the virtual character (e.g., Mario) to jump over obstacles and jump to collect coins by exerting their hand grip, detected by the E-band and transmitted to the computer game to control in-game actions (connected to DAM). The games were also designed to adapt to the current performance of the user (see Figure 5).

To enable this, the game engine tracks the user’s success rate of clearing obstacles over a short period of time (around 15 s). Based on this rate, the game engine automatically makes a decision about whether to increase or decrease the difficulty of the game. Specifically, each game had three difficulty levels. Two characteristics of the game are altered to change the difficulty level: (1) size of obstacles and (2) game speed. In level 1, the character would run at a relatively low rate of speed across the screen and there would only be smaller obstacles to jump over. In level 2, the aforementioned coins are added to level 1. The addition of coins (which should be collected by the player), along with the obstacles (which should be avoided), aims to add an aspect of decision making and increase cognitive load, thereby increasing the difficulty level (level 2) during the gameplay experience; this is part of the wider aim of increasing user engagement. Additionally, the probability of large obstacles generated in the game environment is increased. In level 3, the speed of the game increases further and only large obstacles are generated in the game environment.

**Figure 6:**
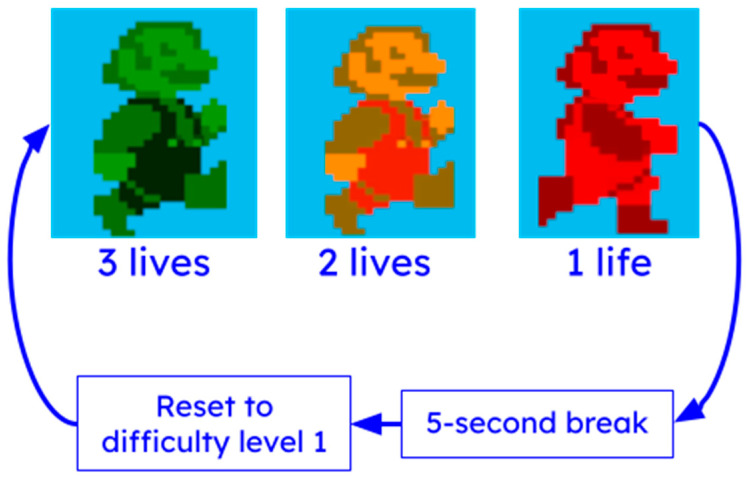
Lives flow structure. The user begins each game with three lives (green color). If they collide with an obstacle, they lose one life, causing the character to change color from green to yellow (two lives), and finally to red (one life) if another collision occurs. If all lives are lost, the game pauses for five seconds and the game difficulty is reset to the lowest level.

Additional tolerance for user error has been incorporated through the use of “lives” (see Figure 6). At the start of each game, the virtual character appears green, indicating that the user has three lives left. If the user fails to jump at the correct time and collides with an obstacle, the virtual character changes color to yellow (two lives remaining), then to red (one life remaining). If the user collides with an obstacle while the virtual character is red, the game pauses for five seconds, and resets the user to the first difficulty level.

Another key component of the game engine is its data-gathering capabilities. During gameplay, the game engine stores data metrics in various spreadsheets for subsequent analysis. These metrics are the (1) timestamps of detected squeeze/clicks output by DAM, (2) timestamps of game difficulty level changes, and (3) timestamps of obstacle clearance or coin collection by player.

### 2.5. Participants

A feasibility study that aimed to determine various metrics of holistic system functionality during its intended use was conducted (see Table 2). All participants provided informed consent and consented to the publication and use of collected data.

Twenty-one participants were recruited and divided into two groups based on whether they self-reported a diagnosis of a neurodegenerative disease (relatively healthy [RH] vs. neurodegenerative disease [ND]). The RH group included 12 participants (age 53.5 ± 25.763 years), and the ND group included 9 participants (age 70.89 ± 10.018 years). Participants in the ND group reported self-diagnoses for PD (4 participants), MS (1 participant), ALS (1 participant), spinal muscular atrophy (SMA; 1 participant), Charcot–Marie–Tooth disease (CMT; 1 participant), and essential tremor (ET; 1 participant). sEMG data for one participant in this group was lost, so it was excluded from all sEMG analyses.

### 2.6. Feasibility Study Protocol

**Figure 7:**
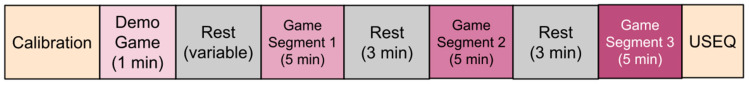
Feasibility study protocol. All participants followed the same protocol, which lasted around 21 min in total.

Participants were recruited using flyers posted across the University of Rhode Island campus, as well as visiting the South County Senior Center, South Kingstown, RI, USA. Once participants expressed interest in participating in the study, the researcher scheduled a prescreening phone call. During this phone call, participants were asked to provide demographic information and to self-report any diagnoses of NDs. The participant’s in-lab study session time was also coordinated on this phone call. Upon arriving at the lab, each participant was given a brief overview of the study protocol (see Figure 7). Then, the participant was instructed to squeeze a stress ball to make it easier to palpate the desired hand flexor muscles of the forearm. A researcher then positioned the E-band with electrodes over the flexor muscle bundle and the reference electrode over the olecranon, tightening it using the Velcro straps to a snug fit. The DAM was attached to the E-band via magnetic connectors (see Figure 3), and the calibration procedure was performed. 

Each participant played a demo game for 1 min to experience the three game environments (see Figure 4) and allowed the researchers to verify that the system was working. After the demo game and a rest period, the participants played the computer game for 15 min total (5 min sessions with 3 min rest periods in between). Finally, participants completed a User Satisfaction Evaluation Questionnaire (USEQ) to gauge their experience with the VRGrip system (see Table 3) [21].

### 2.7. Feasibility Study Data Analysis

For all statistical tests used in this study, we opted for a critical *p*-value of 0.25 because in our exploratory study, using a higher critical *p*-value may allow us to better identify relevant trends, even if the change is small in magnitude. Rather than finding significant differences between groups, which would mean very little at this sample size, our goal with a more liberal critical *p*-value is to identify any potential trends for further study [22]. For example, we aim to characterize muscle fatigue using the collected sEMG data; the literature has shown that a change of 10–15% can be expected in median frequency (MDF) of sEMG signals when muscles are fatigued from maximum volatile contractions [23]. Because participants in this study exerted much less than maximum effort over a relatively short period of time, we expect any trends indicating fatigue to be much smaller in magnitude than the reported 15%; using a larger critical *p*-value may allow us to pick out this trend.

Prior to deeper analysis, the reliability of the VRGrip system in detecting hand grip exertion was determined in both groups by calculating the *F1* score [24]. *F1* score (see Equation (1)) was calculated using true positives (*TP*: participant squeezed and system detected a squeeze), false negatives (participant squeezed, but system did not detect a squeeze), and false positives (participant did not squeeze, but system detected a squeeze). The *F1* score is a measure of the ability of the VRGrip to detect hand grip while accounting for errors caused by *FPs* and *FNs*. A high incidence of *FPs* and *FNs* would result in a low *F1* score. In this context, a low *F1* score would cause participants to have difficulty in controlling the computer game as desired, possibly decreasing user satisfaction and engagement.

(1) $$F1-score=\frac{2TP}{2TP+FP+FN}$$

Perceived exertion of hand grip by the participant was time-marked by researchers by watching the videos recorded during each session. These markers were compared to timestamps of detected jumps collected by the computer game. If a jump was detected by the computer game within 0.2 s of the marked participant squeeze, it was considered a true positive (*TP*). The *F1* scores of the relatively healthy group and the neurodegenerative disease group were compared using a two-sample *t*-test (alpha 0.25).

**Figure 8:**
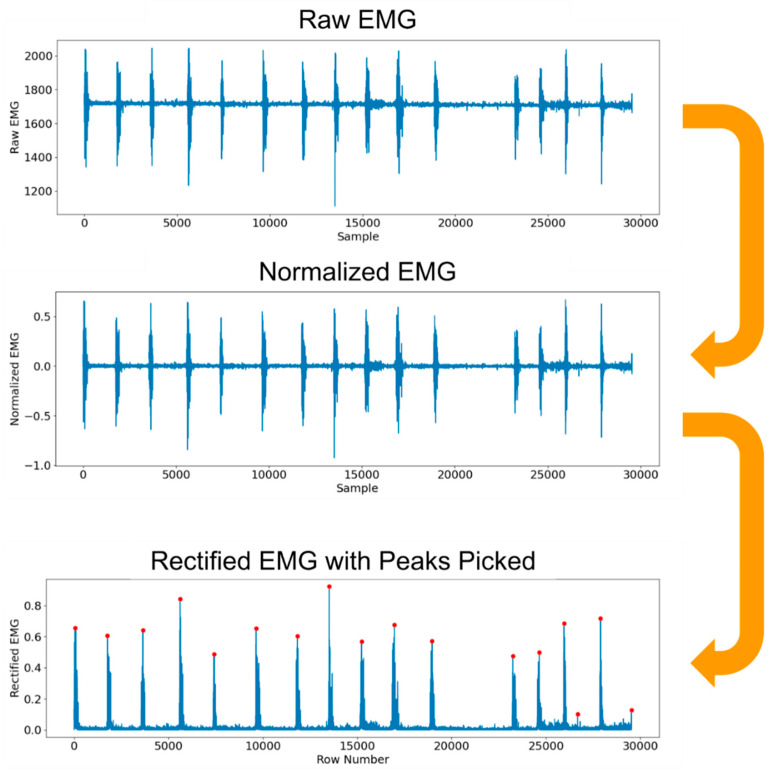
Processed EMG signal samples (feasibility study). The collected EMG data were normalized between −1 and 1 to standardize group comparisons. Normalized data were then rectified, and peaks were picked.

For raw sEMG data collected during the study, frequency analysis was first performed on all datasets. A bandpass filter was used to isolate sEMG frequencies between 20 and 250 Hz, which represents the dominant portion of usable sEMG frequencies [25]. The signals were then normalized between −1 and 1, and subsequently rectified. Then, the Scipy library was used to identify peaks in the sEMG signal that would correspond to grip exertions by the user (see Figure 8) [26]. 

**Figure 9:**
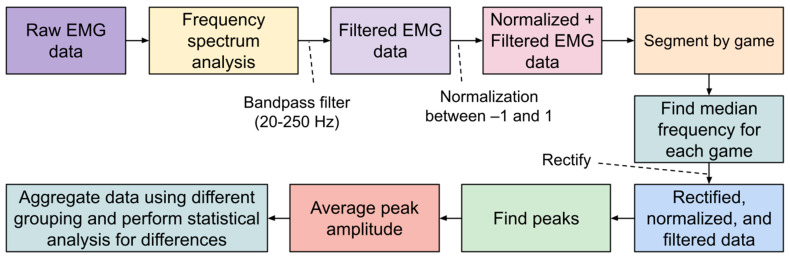
EMG data processing pipeline. All EMG datasets were analyzed using Python 3.11.

We aimed to determine whether or not fatigue is detectable in the collected sEMG. According to the current literature, fatigue can be identified in sEMG by analyzing the wave patterns of the signals. Most commonly, it is seen as a decrease in the median frequency (MDF) of the sEMG signal, though the literature states that varying trends in the amplitude of sEMG signal can also correspond to fatigue. Some studies have shown that a decrease in amplitude corresponds to an increase in fatigue, while others have shown that an increase in amplitude corresponds to an increase in muscle fatigue. For each participant, the collected sEMG data were separated into three distinct segments corresponding to the three games that each participant played. Then, the MDF and mean peak amplitude (MPA) of the signal in each of the three games were determined (see Figure 9).

**Figure 10:**
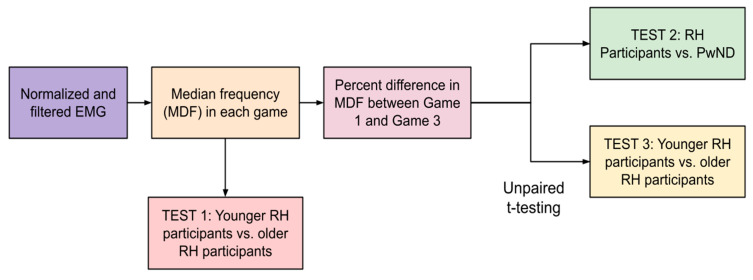
Fatigue analysis pipeline. Using the MDF data obtained from the feasibility study EMG files, we performed three different *t*-tests (Tests 1–3) to determine if fatigue was detectable as described in past literature (i.e., a decrease in median frequency over time as fatigue increases).

These MDF values were used in two ways: (1) to characterize differences in fatigue between relatively healthy participants and participants with neurodegenerative diseases and (2) to characterize differences in sEMG frequency between older and younger relatively healthy participants. The first analysis (Test 1) performed with these data were to determine if older RH participants (75.00 ± 9.32) have lower median frequencies than younger RH participants (32.00 ± 16.25). This would indicate lower muscle fiber recruitment, which is characteristic of older adults [27]. The MDF from all three games was aggregated for younger RH participants and older RH participants and the averages were compared using an unpaired *t*-test (see Figure 10). 

To quantify differences in fatigue between participants during this study, we calculated the percentage difference in the MDF of the sEMG signal between Game 3 and Game 1. The basis of this analysis is the idea that participants would exhibit higher levels of muscle fatigue during the third round of gameplay (between 10 and 15 min of gameplay) than during the first round (between 0 and 5 min of gameplay). 

**Figure 11:**
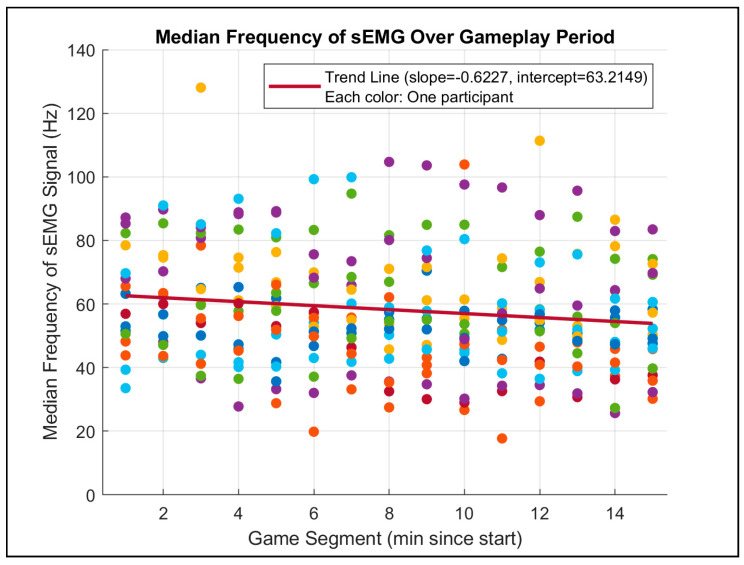
Median EMG frequency change over time. This scatterplot shows a noticeable decrease in MDF over the gameplay period, which is a key indicator of muscle fatigue.

Once percentage differences were obtained for each participant; they were summed by group (RH vs. ND). An unpaired *t-*test was then performed to determine if there were statistically significant differences between the percentage differences in two different comparisons: (1) RH participants vs. ND and (2) younger RH participants (youngest six participants; age 32.00 ± 16.25) vs. older RH participants (remaining participants; age 75.00 ± 9.32)) (see Figure 11). 

## 3. Results of Feasibility Study

### 3.1. VRGrip System Reliability Analysis

**Figure 12:**
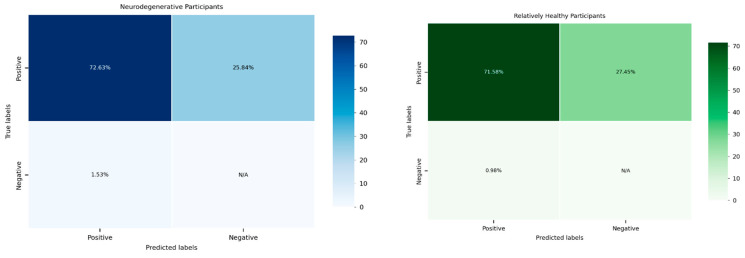
Video-extracted metric confusion matrices. These confusion matrices show the percentage of TPs, FPs, and FNs in both the ND and RH groups. Both groups showed a similar distribution of the metrics.

A statistical analysis was conducted to evaluate the reliability of the system in detecting hand grip exertion across two distinct participant groups: the relatively healthy group and individuals with neurodegenerative diseases (see Figure 12). The calculated F1 scores, a measure of the system’s performance, were subjected to a two-sample *t*-test to determine whether there were significant differences in system reliability between these groups.

The results of the *t*-test revealed a *p*-value of 0.9092, indicating no statistically significant difference between the two groups. This finding suggests that the system demonstrated comparable reliability in detecting hand grip exertion irrespective of the participants’ health conditions. The high *p*-value indicates that any observed differences in F1 scores between the groups were likely due to random variation rather than a systematic disparity in the system’s performance (Table 4).

This finding is promising as it highlights the system’s robustness and its ability to maintain consistent performance across diverse populations. Such consistency is crucial for its application in clinical and non-clinical settings, ensuring equitable reliability regardless of the user’s health status. It also emphasizes the potential of the system to serve as a universal tool for hand grip monitoring, including in populations with neuromuscular impairments. 

### 3.2. VRGrip EMG Signal Analysis

Following the reliability analysis, the raw sEMG recordings obtained from each participant were processed using the predefined signal processing pipeline (refer to Figure 10) to compute the mean peak amplitude (MPA) and median frequency (MDF) of the sEMG signals during each gameplay segment (see Table 5). This detailed analysis aimed to assess the signal attributes and uncover patterns related to muscle activity and fatigue across different participant groups.

Test 1 examined whether there were significant differences in the MDF between older and younger relatively healthy (RH) participants. The results revealed a significant difference (*p* = 0.2315), suggesting that age could have strongly affected the median frequency of sEMG signals in the RH group. Test 2 focused on the comparison of percent change in MDF, a marker of muscle fatigue, between participants with neurodegenerative diseases (ND) and RH participants. The results were not statistically significant (*p* = 0.695), indicating that the system did not consistently differentiate markers of muscle fatigue between these groups. Test 3 evaluated the percentage decrease in MDF in younger and older RH participants and found that older RH participants exhibited a significantly higher fatigue-related decline in MDF compared to younger RH participants (*p* = 0.125). This result aligns with the expectation that aging impacts muscle endurance and fatigue rates. Furthermore, a scatterplot analysis of MDF across 1 min gameplay segments (see Figure 11) demonstrated a consistent decline in MDF over time, which corroborates the previous literature on muscle fatigue during sustained activities.

The analysis also compared the MPA between Game 1 and Game 3 for both RH and PwND participants. Paired *t*-tests showed no significant changes in mean peak values over time for either group, with P-values of 0.9129 and 0.5688, respectively. These findings suggest that the MPA, representing the overall muscle activation amplitude, remained stable throughout the gameplay, irrespective of participant group or game segment. Additionally, no observable trends in MPA were detected over time, indicating that this metric may be less sensitive to fatigue-induced changes compared to MDF.

These findings underscore the potential of MDF as a reliable marker for assessing muscle fatigue, especially in older and/or ND participants, while MPA appeared more consistent and unaffected over the course of gameplay. This dual analysis highlights the complementary nature of these metrics in evaluating neuromuscular performance and fatigue. Future studies could further explore these metrics in larger, more diverse populations to validate these findings and enhance the system’s applicability in clinical and research settings.

### 3.3. User Experience Evaluation of the VRGrip System

The User Experience Questionnaire (USEQ) responses indicate that participants found their interaction with the VRGrip system to be enjoyable, engaging, and responsive (see Table 6). Participants were asked to rate their experience across six key metrics, including enjoyment (Q1), engagement (Q2), system responsiveness (Q3), comfort (Q4), perceived performance (Q5), and ease of use (Q6). Across these categories, responses reflected a generally positive perception of the VRGrip system, with high ratings for enjoyment and engagement, suggesting that the gamified elements of the system successfully held participants’ attention throughout the trial. High scores for responsiveness (Q3) also suggest that the system accurately registered participants’ hand grip efforts, an essential aspect of its functionality.

Free-response feedback (Q7) further highlighted specific areas for system improvement and user observations. Participants suggested the inclusion of resistive balls of different sizes to accommodate varied hand sizes and grip strengths, which could enhance accessibility and user comfort. Additionally, several participants noted that arm fatigue, especially as gameplay progressed, occasionally hindered their ability to maintain a consistent grip effort. This feedback aligns with prior studies indicating that muscle fatigue is a common challenge in prolonged physical interaction tasks, underscoring the need for adaptive gameplay mechanics or rest periods to mitigate fatigue and improve user experience.

These results demonstrate that the VRGrip system effectively engages users while providing an accurate and enjoyable interactive experience. The feedback received provides valuable insights for further refining the system, such as incorporating diverse resistive elements and addressing fatigue-related challenges, to ensure the system’s accessibility and long-term usability across diverse populations. Future iterations should also explore how to dynamically adjust game difficulty or offer real-time feedback to account for muscle fatigue, improving both the system’s effectiveness and the user’s overall experience. Additionally, although the F1 score was satisfactory, the presence of false positives may make the gameplay frustrating for users, who might feel that the game is malfunctioning or reacting randomly. We plan to address this issue by experimenting with different electrode shapes and fabrication techniques, as well as exploring more robust algorithms to detect hand grip from sEMG signals.

## 4. Conclusions

Overall, the results of this preliminary study show that the VRGrip system shows the potential to be an engaging and reliable method to control a virtual environment through the detection of forearm muscle activity associated with hand grip. Analysis of the collected sEMG data during the feasibility study also illustrates that our system can be used to derive EMG-related metrics such as amplitude and frequency. These two metrics can potentially be used for clinical analyses like fatigue detection and differences in muscle fiber recruitment. Our analysis (Test 1) serves as a baseline pilot of the VRGrip system’s ability to perform these tasks because we were able to discern the lower MDF seen in older adults due to lower muscle fiber recruitment, which is a documented trend [25]. Furthermore, findings from Test 3 showed the ability to compare muscle fatigue between groups. The result of Test 3 was interesting, as current literature states that older adults show higher resistance to muscle fatigue than younger adults during intermittent isometric contractions (as performed in this study); however, our study showed no such trend [26]. Further research with larger sample sizes should be conducted to definitively conclude the relationship of this trend.

Participant feedback aligns closely with existing research that shows that using games for rehabilitation increases engagement and may contribute to increased adherence to at-home rehabilitation schedules. However, in contrast to existing works, the VRGrip system is unique as it allows users to receive instant, muscle activity-based feedback during rehabilitation using a compact and wireless wearable device, as opposed to research-grade machines or handheld devices [28,29]. This may make the VRGrip system more suitable for at-home use with minimal setup and specialized equipment. While participants reported that the system was comfortable to use in the USEQ, we aim to collect pressure distribution data in future studies to quantify the amount of pressure the forearm band exerts on the forearm of the participant, which may align with long-term usability.

One limitation of this study arises from the study size and duration. The sample size was relatively small, and the length of the study consisted of only one session with participants only playing the game for fifteen minutes. These limitations may explain the non-significant difference in fatigue between RH participants and ND observed in Test 2. Current literature suggests that people with NDs, particularly PD, display higher levels of muscle fatigue during repeated exertion tasks [30]. Further studies in this area must be carried out to understand the effects a system like ours could have on rehabilitation. To do so, two changes can be made. A longitudinal study, where participants complete several sessions of gameplay over a period of multiple days and participants play for an extended period of time, could be used to determine if regular usage of the VRGrip system for longer durations could improve grip strength or fatigue resistance.

Another limitation of this study is that participants self-reported whether they had a neurodegenerative disorder, which makes it difficult to generalize the results of these studies to any particular disease population. In future studies, we aim to collaborate with clinicians to objectively assess the neurodegenerative disease status of each participant prior to grouping.

In the lab, researchers provided participants with verbal instructions and assistance during many parts of the study session, including calibration and initial gameplay. In an at-home setting, users of the VRGrip system would need to follow instructions provided by the computer game and device to setup the system independently. Future studies need to be conducted in an at-home setting to determine how usable the system is in this at-home setting.

In summary, this work demonstrates that the VRGrip system is not only capable of recording raw sEMG data and integrating it with a computer game but is also enjoyable for participants. In future studies, this technology can be coupled with existing rehabilitation programs that have been prescribed to the patients. Partnering with clinicians will allow us to gain further insight into how a system like VRGrip can fit into a traditional grip rehab protocol. VRGrip can potentially serve as a supplement to the at-home portion of the rehab protocol; patients can play the computer game at home using the E-band and DAM for a duration and frequency defined by the clinician. A secure portal can also be built such that clinicians can access the EMG recordings and game performance of each patient for analysis over time. This integration will allow clinicians to have objective data about what the patients are doing for rehabilitation at home.

While this study only included participants with mild-to-moderate NDs (self-reported symptomatology), the effectiveness of the VRGrip system to detect hand grip should be assessed in participants with more severe symptoms like muscle tremors. Muscle tremors are particularly interesting because VRGrip relies on muscle activity for hand grip detection, and the periodic impulses generated by muscle tremors could pose a challenge to our calibration/detection algorithms. Our system’s use in individuals with more severe physical symptoms may also warrant a redesign of the device to increase wearability and comfort.

The application of this type of intervention could extend well beyond finger flexion, potentially focusing on fine motor development in the hand and wrist or expanding to other parts of the body to provide sEMG-based movement rehabilitation or training. This work shows possible technological advancements that could potentially enhance traditional rehabilitation in an era of technological progress and increase digital health awareness; when an estimated 1 in 3 individuals live with conditions that could benefit from physical rehabilitation, devices like the VRGrip system could revolutionize rehabilitation practices [31].

To contextualize the need for VRGrip, we must compare our system to past sEMG-based upper limb rehabilitation approaches. For example, Oyemakinde et al. designed a relatively discreet sensor module that combines both sEMG and FMG (force myography: measuring the change in muscle shape during contraction through pressure sensors) [32]. During the study, ten participants performed fifteen different hand gestures while EMG and FMG were being recorded. Later, the data were classified into the different hand gestures using a convolutional neural network (CNN). The CNN was extremely effective at classifying these gestures, with a 95% accuracy rate. However, this system suffers from two major drawbacks: (1) the entire system was wired, with one wire running from each EMG electrode to a bioinstrumentation device, and (2) the classification of hand gestures was not done in real time. VRGrip addresses these concerns by using the DAM to collect sEMG signals and transmit them wirelessly, which reduces the bulk of wires and the need for cable management. Additionally, the VRGrip system is capable of performing real-time recognition of hand gestures, albeit only one gesture at this time, making it more viable for interactive hand grip rehabilitation than the CNN-based classification described by Oyemakinde et al.

Dash et al. directly targeted hand rehabilitation using sEMG and a VR game system called “Gripx” [33]. The study involved 20 participants (12 post-stroke, 8 healthy). Adhesive sEMG electrodes were placed on the forearms of the participants and the electrodes were connected by wire to a tabletop sEMG-VR handshake device that transduced the collected sEMG data to inputs for the VR game. The post-stroke participants were exposed to the system upwards of 10 times each over the course of multiple days, and exhibited an increase in game-specific performance, as well as hand grip force, as measured by a dynamometer. The strength of this study lies in displaying the potential for sEMG-based hand grip rehabilitation tools to improve hand grip strength in post-stroke individuals. While the VRGrip system has not been shown to improve hand grip strength over time, we aim to conduct longitudinal studies that could answer this question, similarly to Dash et al. VRGrip differs from the system used in this study because our sEMG acquisition and handshaking device are combined into one device, the DAM, which directly connects to our forearm band.

Compared to the systems used in these studies, the VRGrip system also has a major advantage: the use of e-textile electrodes rather than metal or adhesive sEMG electrodes. Because we aim to test the VRGrip system for long-term at-home use, e-textile electrodes can present a comfortable alternative to other types of electrodes, which can cause skin irritation or injury. The small size and portability of the VRGrip system also makes it well-positioned for at-home use, compared to the other studies that use lab-grade machines for data acquisition. However, the above studies also have interesting aspects that we aim to incorporate in the future, namely the use of a CNN to classify sEMG features and a longitudinal study to analyze the effects of using the VRGrip system on hand grip strength.

## Figures and Tables

**Table 1:** Indicator LED color chart.

Status Code	LED State	LED Color
S1	Standby State	Red
S2	Relax Grip State	Yellow
S3	Maximal Grip Squeeze State	Blue
S4	Calibrated state	Green

**Table 2:** Participant demographics.

Participant ID	Age	Self-Reported Diagnosis
P1	19	RH
P2	19	RH
P3	23	RH
P4	26	RH
P5	49	RH
P6	56	RH
P7	63	RH
P8	69	RH
P9	72	RH
P10	75	RH
P11	82	RH
P12	89	RH
ND1	61	PD
ND2	78	PD
ND3	79	PD
ND4	81	PD
ND5	61	MS
ND6	66	ALS
ND7	57	SMA
ND8	71	CMT
ND9	84	ET

**Table 3:** User Satisfaction Evaluation Questionnaire.

Q1	How much did you enjoy the experience with the VRGrip sleeve?
Q2	How engaged were you with the computer game?
Q3	How effective do you feel the system was at responding accurately when you squeezed?
Q4	How comfortable was the overall experience?
Q5	Do you feel like you did a good job squeezing throughout the trial?
Q6	Did you feel the system was simple and easy to use?
Q7	Please provide any feedback about your experience. (free response)

**Table 4:** Video-extracted metrics.

Group	TP	FP	FN	F1 Score
RH	3450	1323	47	0.8343 ± 0.1208
ND	2184	777	46	0.8401 ± 0.1034

**Table 5:** EMG signal attributes: MPA—mean peak amplitude; MDF—median frequency.

	MPA (Normalized −1 to 1)			MDF (Hz)		
Group	Game 1	Game 2	Game 3	Game 1	Game 2	Game 3
RH	0.4079 ± 0.1850	0.3928 ± 0.1874	0.4129 ± 0.2155	59.9068 ± 13.6648	55.4616 ± 19.9541	52.6693 ± 16.7540
ND	0.3316 ± 0.2745	0.3411 ± 0.2889	0.3663 ± 0.2699	63.8200 ± 18.7389	60.8666 ± 14.0662	59.5703 ± 17.0589

**Table 6:** Aggregated USEQ scores.

	Mean Response ± SD
Q1	4.76 ± 0.44
Q2	4.81 ± 0.40
Q3	4.05 ± 0.97
Q4	4.95 ± 0.22
Q5	4.38 ± 0.80
Q6	4.95 ± 0.21

## Data Availability

The datasets generated and analyzed during the study are available from the corresponding author on reasonable request.
